# Multivariate linear-mixed analysis of changes in anterior inferior iliac spine impingement incidence with posterior pelvic tilt: a computer simulation study

**DOI:** 10.1093/jhps/hnae003

**Published:** 2024-02-01

**Authors:** Emi Kamono, Naomi Kobayashi, Yuya Yamamoto, Yohei Yukizawa, Hideki Honda, Hyonmin Choe, Hiroyuki Ike, Ken Kumagai, Yutaka Inaba

**Affiliations:** Department of Orthopaedic Surgery, Yokohama City University Medical Center, 4-57 Urafune-cho, Minami-ku, Yokohama, Japan; Department of Orthopaedic Surgery, Yokohama City University Medical Center, 4-57 Urafune-cho, Minami-ku, Yokohama, Japan; Department of Orthopaedic Surgery, Yokohama City University Medical Center, 4-57 Urafune-cho, Minami-ku, Yokohama, Japan; Department of Orthopaedic Surgery, Yokohama City University, 3-9 Fukuura, Kanazawa-ku, Yokohama, Japan; Department of Orthopaedic Surgery, Yokohama City University Medical Center, 4-57 Urafune-cho, Minami-ku, Yokohama, Japan; Department of Orthopaedic Surgery, Yokohama City University Medical Center, 4-57 Urafune-cho, Minami-ku, Yokohama, Japan; Department of Orthopaedic Surgery, Yokohama City University, 3-9 Fukuura, Kanazawa-ku, Yokohama, Japan; Department of Orthopaedic Surgery, Yokohama City University, 3-9 Fukuura, Kanazawa-ku, Yokohama, Japan; Department of Orthopaedic Surgery, Yokohama City University, 3-9 Fukuura, Kanazawa-ku, Yokohama, Japan; Department of Orthopaedic Surgery, Yokohama City University, 3-9 Fukuura, Kanazawa-ku, Yokohama, Japan

## Abstract

It is well known that increased posterior tilt of the pelvis is an effective strategy for avoiding impingement of the femur with the pelvis during movement. Daily repetitive collisions become mechanical loads, and the more frequently they occur, the more tissue damage and pain they cause. Therefore, reducing the rate of occurrence of impingement is important to avoid aggravation of symptoms. This study aimed to evaluate the effects of changes in posterior pelvic tilt on the risk of impingement between the femur and the anterior inferior iliac spine (AIIS)/subspine in various functional postures. Patients with femoroacetabular impingement syndrome (FAIS) who were candidates for hip arthroscopic osteochondroplasty between October 2013 and June 2020 were included. A three-dimensional reconstructed model was used to simulate the incidence of impingement at 12 hip positions required for activities of daily living. We predicted value of the spatial incidence of impingement assumed that hip motion should exceed 130/30 degrees without impingement. Impingement was measured at three pelvic positions: an anterior tilt of 10°, in the functional pelvic plane and a posterior tilt of 10°. Multivariate linear-mixed models were used to assess the effect of covariate-adjusted posterior pelvic tilt on the impingement incidence in the AIIS region. AIIS type, center-edge angle, acetabular version and femoral version were used as covariates. The impingement rates and locations of the three pelvic tilt postures were assessed. Seventy-eight patients (60 males and 18 females; average age, 46 ± 15.1 years) with FAIS were analyzed. A multivariate linear-mixed model revealed a coefficient of −0.8% (95% confidence interval −0.9 to −0.7%; *P* < 0.001) for posterior pelvic tilt. Thus, posterior pelvic tilt affects AIIS impingement incidence. After adjusting for anatomical effects, the posterior pelvic tilt should be addressed to avoid impingement.

## INTRODUCTION

Impingement between the femur and the anterior inferior iliac spine (AIIS) or the pelvic subspine is common in individuals with femoroacetabular impingement syndrome (FAIS)[1]. Problems arise because of frequent collisions between the soft tissues (joint capsule, rectus femoris tendon and labrum) that lie between the AIIS and femur [2]. Athletes who move the hip joint in a complex pattern (e.g. soccer players), or who flex the hip joint excessively (e.g. ballet dancers and other dancers), often develop this condition [3]. AIIS impingement commonly occurs alongside FAIS and is a common cause of failed hip preservation surgery, requiring revision due to insufficient FAI resection [4]. Therefore, avoiding impingement in this region is necessary to avoid poor post-operative prognosis.

The primary treatments for FAIS are conservative treatment and physiotherapy-led rehabilitation; hip arthroscopy should be considered for non-responders [5]. A previous systematic review and meta-analysis of patients with FAIS indicated that conservative interventions result in beneficial outcomes in terms of reduced pain and improved function [6]. To consider effective conservative treatments, it is necessary to understand the physiological mechanisms that influence the impingement of AIIS regions. Patients with FAIS exhibited less pelvic tilt during asymmetric hip flexion [7]. A previous study used a simulation model to show that changing the pelvic tilt is effective in reducing impingement [8]. Although there is no conclusive evidence to suggest that avoiding impingement in specific detailed regions prevents pain or improves postoperative outcomes, we hypothesized that dynamic pelvic tilt may have a significant effect on impingement incidence in the AIIS region.

The anatomy is an important factor in the pathogenesis of FAIS. Pelvic and femoral geometries, such as femoral version (FV) and center-edge (CE) angles, are known predictors of the symptomatic femoroacetabular impingement [9, 10], and their influence cannot be ignored. Frequent impingement in the AIIS region may be related to its anatomy [1]. Multiple anatomical factors can be confounding when exploring the causal relationship between impingement and pelvic tilt. Therefore, we used a regression model adjusted for morphological factors to analyze the effect of pelvic tilt on impingement.

This study aimed to use a multivariate linear-mixed model, with hip anatomy as a covariate, to examine the effect of changes in pelvic tilt on the incidence of AIIS/subspine impingement.

## METHODS

This case series was approved by our Institutional Review Board. The inclusion criteria were as follows: patients with FAIS who were candidates for primary arthroscopic hip osteochondroplasty (cam, pincer or combined resection) between October 2013 and June 2020. Exclusion criteria included previous rotational acetabular osteotomy (RAO) and missing computed tomography (CT) images. The diagnosis of FAIS was based on the radiological data. Cam-type FAIS was defined as an alpha angle > 55° on a cross-table lateral view or 45° on a flexion Dunn view [11, 12]. The pincer-type FAIS was defined as a lateral center-edge angle (LCEA) > 40° on the anteroposterior view of the pelvis [13] and combined-type FAIS was defined as the presence of both cam and pincer deformities. Diagnostic criteria did not change during the study period.

### Computer simulation model

**Fig. 1.**
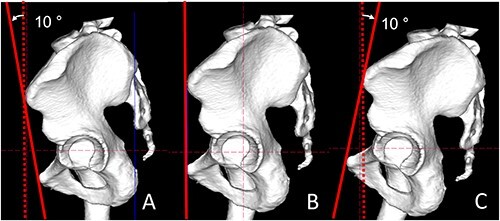
Image viewed from the left in the sagittal plane. Three pelvic model conditions. (A) A 10° anterior tilt (solid line) with respect to the dotted line (FPP). (B) The FPP. (C) A 10° posterior tilt (solid line) with respect to the dotted line (FPP).

All CT scans (Siemens AG) were performed using a standardized protocol at a single institution. Pelvic and femur CT scans, including those of the femoral condyle, were performed using 1.5-mm-thick slices. Scans were performed with the patient in the supine position. The 3D reconstruction and dynamic study were performed using ZedHip® (Lexi, Tokyo) software. The ZedHip is a preoperative planning medical device that has been validated and approved by the Pharmaceuticals and Medical Devices Agency (approval number:227AABZX00075000). This study used the functional pelvic plane (FPP) that considers pelvic tilt of the pelvis [13]. FPP was defined by the bilateral anterior superior iliac spine and the *Z*-axis of the CT coordinate [14]. For the femoral plane, reference points around the femoral head in the axial and sagittal planes were used to define the femoral head center. Points on the medial/lateral epicondyles and posterior condyles, knee center, greater trochanter tip and lesser trochanter were also identified. The following three factors were set as conditions for the pelvic model: viewed in the FPP, 10° anterior pelvic tilt and 10° posterior pelvic tilt (Fig. 1). The morphology of the AIIS was assessed in accordance with the classification of Hetsroni [15], using 3D reconstruction data.

### Definition of impingement

The collision points were identified using a 3D dynamic simulation created using a Zed Hip. In this system, impingement was defined as the impact between the pelvis and femur.

**Fig. 2.**
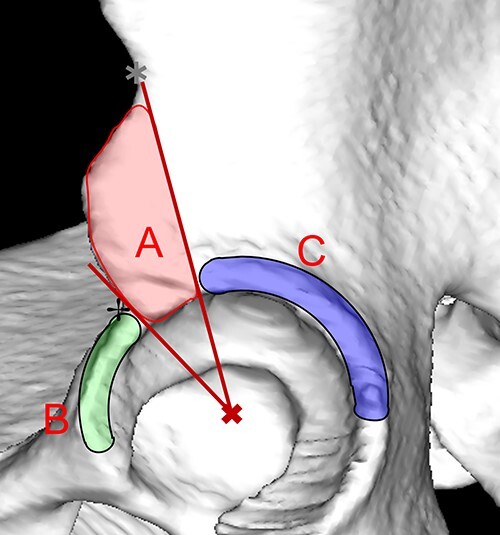
Image viewed from the left in the sagittal plane. Definition of the impingement site. (A) The AIIS/subspine region surrounded by the following two lines in the sagittal plane: the line passing through the inflection point (asterisk) between the anterior superior iliac spine and AIIS and the center of the acetabular rim, and the line passing through the inflection point (dagger) between the AIIS and anterior acetabular rim and the center of the acetabulum. (B) Acetabular rim region anterior to (A). (C) Acetabular rim region posterolateral to (A).

The location of the impingement region on the acetabular rim or AIIS/subspine region was defined as shown in Fig. 2. The region was specified using anatomical markers according to a previously described clock system [16]. The AIIS/subspine region (A) was defined as the acetabular region bounded by the following two lines in the lateral view just above the acetabular plane: a line passing through the inflection point between the anterior superior iliac spine and AIIS and the center of the acetabulum, and a line passing through the inflection point between the AIIS and the anterior acetabular rim and the center of the acetabulum. The acetabular rim region anterior to A was defined as the anterior region (B), and the acetabular rim region posterolateral to A was defined as the posterolateral region (C).

### Measurement of outcome

The primary outcome was the impingement incidence. Based on previous studies, the maximum range of motion (ROM) required for activities of daily living (ADL) was set at 130° and 30° for flexion and internal rotation, respectively [17, 18]. The occurrence and location of AIIS impingement at the maximum hip flexion or maximum hip internal rotation under 12 hip conditions (Table I) were measured. To measure maximum internal rotation, internal rotation was increased from nine baseline postures, which were a combination of three flexion patterns and three adduction patterns, until bone impingement was detected. The hip flexion patterns were 90°, 70°, and 45°, which are necessary for daily activities (squatting [19], climbing stairs [20], and walking [21]).

**Table I.** Definition of the presence of impingement and 12 hip postures

Maximum hip flexion is <130°
➢ At 0° of adduction
➢ At 10° of adduction
➢ At 20° of adduction
Maximum internal rotation angle is <30° at 90° flexion
➢ 0° of adduction
➢ 10° of adduction
➢ 20° of adduction
Maximum internal rotation angle is <30° at 70° flexion
➢ 0° of adduction
➢ 10° of adduction
➢ 20° of adduction
Maximum internal rotation angle is <30° at 45° flexion
➢ 0° of adduction
➢ 10° of adduction
➢ 20° of adduction

To measure maximum internal rotation, two variables, flexion and adduction, were fixed, and the third variable (internal rotation) was increased until bone impingement was detected. To investigate the incidence of impingement in various daily life situations, we measured the incidence of impingement occurrence in 12 situations. Impingement incidence was calculated as follows: incidence of impingement = number of impingements at each location/12 (number of conditions) × 100.

The primary endpoint was the incidence of impingement in the AIIS/subspine region in the three pelvic postures. The secondary endpoint was the incidence of impingement in the three acetabular regions.

In addition, the maximum angle for each posture at each posterior tilt angle of the pelvis was calculated.

### Statistical analysis

Continuous variables were calculated as the mean ± standard deviation [with 95% confidence intervals (CI)]. Categorical data were calculated by aggregation. The effect of the posterior pelvic tilt on impingement incidence was evaluated using a multivariate linear mixed model. Deviance, conditional Akaike information criterion (cAIC) and Bayesian information criterion (BIC) were used for model comparison. We used a restricted maximum likelihood estimation to avoid bias in the variance estimator of the random effects. The AIIS type, CE angle and FV were included as covariates. The patients were treated as random intercepts. The Satterthwaite approximation was used for a two-sample *t*-test of the degrees of freedom. A *P*-value < 0.05 was considered to be statistically significant. All statistical analyses were performed using R version 4.1.2 (R Foundation for Statistical Computing, Vienna, Austria) [22].

## RESULTS

**Fig. 3.**
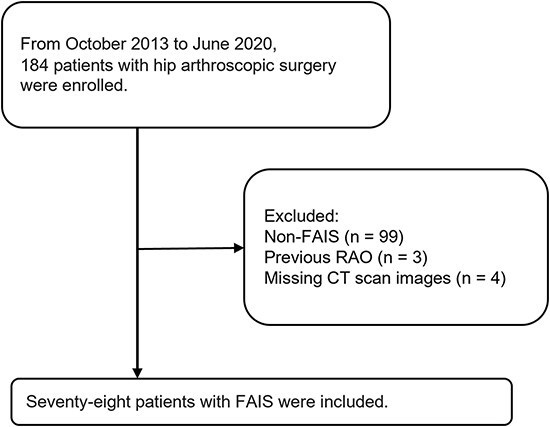
Patient selection flow chart.

Of the 147 patients who underwent arthroscopic surgery during the study period, 78 who were diagnosed with FAIS and underwent hip arthroscopic osteochondroplasty were analyzed (Fig. 3). The average age of the patients was 46 years (18 females and 60 males). The average FV was 14.19 ± 10.84°. There were 71 hips with a cam deformity, two hips with a pincer deformity and five hips with a combined deformity. Sixteen hips were classified as type I and 12 as type II (Table II).

RAO, rotational acetabular osteotomy.

**Table II.** Preoperative patient characteristics and radiographic parameters

	Patients (*n* = 78)
Sex, *n*	
Male	60
Female	18
Age, years	46 ± 15.1
BMI, kg m−^2^	23.9 ± 3.8
Femoral version (CT), deg	14.19 ± 10.84
Femoral version type, *n*	
Low (<5°)	19
Normal (5–19°)	24
High (>19°)	35
Acetabular version (CT), deg	18.63 ± 6.34
LCEA (radiograph), deg	30.77 ± 6.55
LCEA type, n	
Low (<25°)	12
Normal (25–40°)	15
High (>40°)	51
Alpha angle (CT), deg	67.13 ± 8.89
FAI type, *n*	
Cam-type FAI	71
Pincer-type FAI	2
Combined-type FAI	5
Diagnosis, *n*	
BDDH	13
OA	4
Other	2
AIIS type, *n*	
Type I	28
Type II	50
Type III	0

Data are presented as the mean ± SD unless otherwise stated.

BMI, body mass index; deg, degrees; *n*, number.

The multivariate linear-mixed effects regression model was the best-fit model because it had the lowest cAIC, BIC and deviance values (Table III).

**Table III.** Model comparison between standard regression and mixed effects regression

Model comparison	cAIC	BIC	Deviance
Univariate-mixed effect model			
AIIS type	−156.8	−143.2	−155.3
Femoral Version	−167.3	−153.7	−159.0
Posterior Pelvic Tilt	−248.7	−235.0	−238.5
LCEA	−154.9	−141.3	−148.2
Multivariate regression model	−64.7	−41.1	−79.28
Multivariate-mixed effect regression model	−259.4	−232.4	−276.1

cAIC, conditional Akaike information criterion; BIC, Bayesian information criterion.

The multivariate linear-mixed model identified the posterior pelvic tilt (*P* < 0.001) and femoral version (*P* < 0.001) as significant factors influencing impingement incidence. The coefficient of pelvic tilt was −0.8% (95% CI, −0.9 to −0.7%; *P* < 0.001). The coefficient of FV was −0.7% (95% CI, −1.0 to −0.3%) (Table IV).

**Table IV.** Multivariate linear-mixed model examining changes in impingement rate at the AIIS/subspine

			95% CI	
Factor	Β value (%)	P-value	Lower	Upper
Posterior pelvic tilt	−0.8	<0.001	−0.9	−0.7
AIIS type = I	6.7	0.166	−16.3	2.9
AIIS type = II	0	.	.	.
Femoral version (CT)	−0.7	<0.001	−1.0	−0.3
Acetabular version (CT)	0.06	0.819	−0.6	0.8
LCEA	0.6	0.101	−0.1	1.3

FV, femoral version.

The average total impingement frequencies were 68.9 ± 23.4%, 56.2 ± 27.0% and 44.7 ± 27.5% at 10° anterior pelvic tilt, in the FPP and at 10° posterior pelvic tilt, respectively (Table V). The impingement incidence was assessed at each setting for each region, and the AIIS/subspine region was found to be the most common site. The average frequencies of impingement were 42.1 ± 24.7%, 33.5 ± 23.2% and 26.4 ± 20.9% at 10° anterior pelvic tilt, in the FPP and at 10° posterior pelvic tilt, respectively. The next most common region was the anterior region: impingement frequencies at 10° anterior pelvic tilt, in the FFP and at 10° posterior pelvic tilt were 16.0 ± 21.5%, 13.7 ± 18.3% and 13.2 ± 17.6%, respectively. Posterior region impingement frequencies at 10° anterior pelvic tilt, in the FFP and 10° posterior pelvic tilt were 9.1 ± 16.9%, 8.5 ± 17.4% and 4.9 ± 12.3%, respectively.

**Table V.** Incidence of impingement at the three regions

	10°of anterior pelvic tilt		FPP		10°of posterior pelvic tilt	
Total impingement (%)	68.9	± 23.4	56.2	± 27.0	44.7	± 27.5
Details of region (%)						
Anterior	16.0	± 21.5	13.7	± 18.3	13.2	± 17.6
AIIS/subspine	42.1	± 24.7	33.5	± 23.2	26.4	± 20.9
Posterolateral	9.1	± 16.9	8.5	± 17.4	4.9	± 12.3

Data are presented as the mean ± SD.

Table VI shows the results of the subgroup analysis by comorbidities and anatomical factors.

**Table VI.** Subgroup analysis of impingement incidence for anatomical factors

	Patients (*n*)		10° of anterior pelvic tilt				FPP				10°of posterior pelvic tilt	
BDDH	13		53.2		± 24.9		37.2		±29.6		26.9	±31.6
AIIS type												
Type I	28		76.7		± 21.4		66.4		± 27.6		53.3	± 27.3
Type II	50		64.6		± 23.4		50.5		± 25.2		39.8	± 26.6
FV type												
Low (<5°)	19		79.2		± 20.0		66.7		± 24.9		55.9	± 29.7
Normal (5–19°)	24		70.0		± 21.4		56.7		± 26.0		44.5	± 24.5
High (>19°)	35		53.9		±24.1		42.1		±26.4		30.7	± 24.2
LCEA												
Low (<25°)	12		53.3		± 24.4		38.3		± 29.7		29.4	± 29.9
Normal (25–40°)	15		73.0		± 20.5		62.1		± 23.9		48.9	± 29.6
High (>40°)	51		70.8		± 27.4		53.4		± 28.1		45.8	± 32.7

BDDH, borderline developmental dysplasia of hip.

**Fig. 4.**
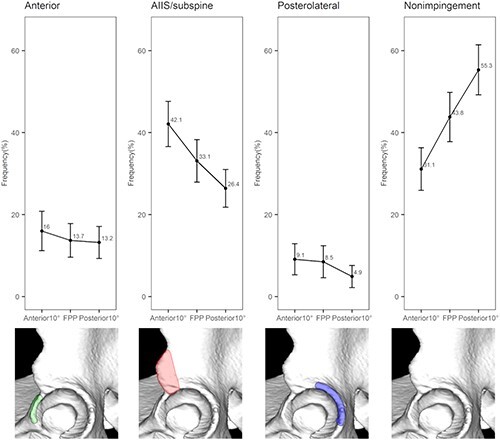
Impingement incidence at the three acetabular regions under three pelvic model conditions. (A) Incidence of impingement at the anterior region. (B) Incidence of impingement at the AIIS/subspine region. (C) Incidence of impingement at the posterior region. The diamond denotes the average value, and the error bars represent the 95% CIs.

Figure 4 shows line graphs of the incidence of impingement in each region according to the change in pelvic tilt. Significant changes in impingement incidence at the AIIS/subspine region were observed with changes in pelvic position (Fig. 4).

## DISCUSSION

The most important finding of the current study was that AIIS/subspine impingement can be avoided by increasing the posterior pelvic tilt regardless of the AIIS shape, FV or CE angle for the ROM required for daily living. This finding is clinically important because it indicates that effective physiotherapy may reduce the incidence of AIIS/subspine region impingement.

In our study, we found that posterior pelvic tilt reduced the incidence of impingement in the AIIS/subspine region. Our linear model showed a 0.8% decrease in impingement incidence with a 1° change in the posterior pelvic tilt. Previous findings on posterior pelvic tilt associated with hip flexion have reported that it contributes ∼8.1% of the posterior pelvic tilt to unilateral hip flexion, and the posterior pelvic tilt at maximum hip flexion was 19.1° in the standing position [23]. Coordinated movements of the pelvis and femur, known as pelvifemoral rhythm, significantly affect the symptoms of patients with FAIS. The posterior pelvic tilt in the sitting position was 3.9° lower in the symptomatic FAIS group than in the asymptomatic FAIS group [23]. Lamontagne observed that patients with FAIS were no different from a control group with respect to hip motion during squatting; however, they had a decreased range of pelvic motion in the sagittal plane (14.7 ± 8.4° versus 24.2 ± 6.8° in controls) [24]. Simulation studies have shown that posterior pelvic tilting is an effective treatment option for FAIS [8]. Applying the abovementioned values to the model of this study, the incidence of impingement occurring in various ADL, such as sitting and squatting, was 3–10% higher in the FAIS.

Anatomically, a decreased FV has been identified as a risk factor for poor outcomes in patients after conservative arthroscopic surgery [1]. The location and incidence of impingement depends on the contact site on the femoral side, and a reduced FV leads to greater contact with the anterior greater trochanter. Subgroup analyses by covariates were performed but failed to account for large standard deviations. In terms of model fit, the cAIC, BIC and deviance values of the univariate regression model were greater than those of the multivariate linear regression model with pelvic tilt, AIIS type, FV and CE as covariates, and the mixed regression model had the lowest cAIC, BIC and deviance values. Model fitting accounted for individual anatomical features as factors and patient variability that could not be explained by covariates influencing the incidence of impingement. Our multivariate linear–mixed effects analysis provided evidence that reduced FV and anterior pelvic tilt are risk factors for impingement incidence in the AIIS/subspine region.

A previous computer simulation analysis that evaluated the distribution of impingement points on the acetabular side showed that the most common impingement point was in the 1–2 o’clock region, just below the AIIS/subspine [25]. These findings are consistent with the labral injury site observed during arthroscopic surgery [26]. Similar results were obtained in our study. Since the AIIS/subspine region is the attachment site for the rectus femoris muscle, impingement around that region is related to extra-articular groin pain [24]; it has been observed that AIIS/subspine impingement may also affect the severity of intra-articular symptoms and lead to a poor post-operative clinical outcome [27]. Arthroscopic findings after surgery to repair a damaged labrum showed that patients with AIIS impingement are much more likely to have a complex labral tear necessitating labral reconstruction [28]. Furthermore, AIIS/subspine impingement is one of the main causes of revision after hip arthroscopic surgery [4]. Thus, we should be mindful of the importance of AIIS/subspine morphologies affecting extra-articular and intra-articular pathologies.

Computer simulations revealed that bony impingement necessarily occurs at the maximum ROM, even though this may be an unrealistic position in real life. Therefore, the model cannot assess realistic impingement incidence during daily activities. Hence, we defined the occurrence of impingement not as the maximum ROM but rather as the estimated ROM required for ADL [17] and assessed individuals with or without impingement within that ROM. According to a cadaver study, internal rotation at 45° hip flexion is limited by soft tissues [29]. Hara et al. used a 3D-to-2D model-to-image registration technique to evaluate the kinematics of healthy hips during gait, squatting, rising from a chair and twisting. When patients performed these four movements, they observed a maximum hip flexion angle of 108.5 ± 13.2°, a maximum hip adduction angle of 6.4 ± 4.9°, and a maximum hip internal rotation angle of 29.2 ± 13.5° [17]. In contrast, Catelli reported that the ROM during hip flexion while deep squatting (approximately 130°) was larger than that reported by Hara [18]. The results of these studies indicate that ROM is not limited solely by bony impingement and that the maximum ROM at which impingement occurs during computer simulation is not always required for ADL. This measurement method was adopted in the current study to compensate for the limitations of a computer simulation based on a bone model and to obtain clinically meaningful results by measuring the incidence of impingement within a practical daily ROM.

This study has several limitations. First, it was based on simulations using CT models and did not consider the effects of actual motion. Therefore, patients should undergo physiotherapy to promote pelvic tilt mobility, followed by verification of its clinical effects. Second, an algorithm with a single center of rotation for hip ROM simulation can be used. To simulate the ROM for complex deformities, including protrusio acetabuli and acetabular retroversion, classic motion algorithms with a single center of rotation have demonstrated limitations and can be unreliable [30]. Furthermore, the collision-detection software only calculates the ROM for bones and does not consider soft tissues (lips, muscles or cartilage). This is a well-known limitation of computer simulations of hip CT-based ROM [31]. Han et al. reported that ROM was overestimated when soft tissue effects were ignored [29]. Therefore, it is feared that the occurrence incidence of impingement is actually higher than that in our study. Third, CT was performed in the supine position. It has been shown that the supine position has an anterior pelvic tilt of 7.59° compared to the standing position [32]. In our survey, it is possible that the measurement was performed with a more anterior pelvic tilt. However, in this study, we measured it in the range of 20°, and because the effect of avoiding impingement of the posterior pelvic tilt was the same from any position, it is inferred that similar results could be obtained in the standing position. This position may lead to an increased pelvic tilt at baseline. The defined ROM required for daily activity was based on previous studies that did not assume sports activities involving high kicking (e.g. soccer or ballet dancing). Those undertaking high-kicking sporting activities are at a high risk of AIIS/subspine impingement [3]. Therefore, our data on the incidence of AIIS/subspine impingement may underestimate its occurrence in populations undergoing high-kicking sports activities.

In conclusion, we revealed the impact of AIIS impingement incidence on posterior pelvic tilt after adjusting for morphological effects. These data suggest that a posterior pelvic tilt reduces the risk of impingement in the region around the AIIS/subspine.

## Data Avaiilability

The data underlying this article will be shared upon reasonable request from the corresponding author.
